# Description of a Nanobody-based Competitive Immunoassay to Detect Tsetse Fly Exposure

**DOI:** 10.1371/journal.pntd.0003456

**Published:** 2015-02-06

**Authors:** Guy Caljon, Shahid Hussain, Lieve Vermeiren, Jan Van Den Abbeele

**Affiliations:** 1 Unit of Veterinary Protozoology, Department of Biomedical Sciences, Institute of Tropical Medicine Antwerp (ITM), Antwerp, Belgium; 2 Unit of Cellular and Molecular Immunology, Vrije Universiteit Brussel (VUB), Brussels, Belgium; 3 Laboratory of Myeloid Cell Immunology, VIB, Brussels, Belgium; 4 Laboratory of Zoophysiology, Department of Physiology, University of Ghent, Ghent, Belgium; IRD/CIRDES, BURKINA FASO

## Abstract

**Background:**

Tsetse flies are the main vectors of human and animal African trypanosomes. The Tsal proteins in tsetse fly saliva were previously identified as suitable biomarkers of bite exposure. A new competitive assay was conceived based on nanobody (Nb) technology to ameliorate the detection of anti-Tsal antibodies in mammalian hosts.

**Methodology/Principal Findings:**

A camelid-derived Nb library was generated against the *Glossina morsitans morsitans sialome* and exploited to select Tsal specific Nbs. One of the three identified Nb families (family III, TsalNb-05 and TsalNb-11) was found suitable for anti-Tsal antibody detection in a competitive ELISA format. The competitive ELISA was able to detect exposure to a broad range of tsetse species (*G. morsitans morsitans*, *G. pallidipes*, *G. palpalis gambiensis* and *G. fuscipes*) and did not cross-react with the other hematophagous insects (*Stomoxys calcitrans* and *Tabanus yao*). Using a collection of plasmas from tsetse-exposed pigs, the new test characteristics were compared with those of the previously described *G. m. moristans* and rTsal1 indirect ELISAs, revealing equally good specificities (> 95%) and positive predictive values (> 98%) but higher negative predictive values and hence increased sensitivity (> 95%) and accuracy (> 95%).

**Conclusion/Significance:**

We have developed a highly accurate Nb-based competitive immunoassay to detect specific anti-Tsal antibodies induced by various tsetse fly species in a range of hosts. We propose that this competitive assay provides a simple serological indicator of tsetse fly presence without the requirement of test adaptation to the vertebrate host species. In addition, the use of monoclonal Nbs for antibody detection is innovative and could be applied to other tsetse fly salivary biomarkers in order to achieve a multi-target immunoprofiling of hosts. In addition, this approach could be broadened to other pathogenic organisms for which accurate serological diagnosis remains a bottleneck.

## Author Summary

Our previous studies have revealed that the saliva of the savannah tsetse fly (*Glossina morsitans morsitans*) and the main constituting Tsal proteins are sensitive immunological probes to detect contact with tsetse flies. A nanobody (Nb) library was generated against tsetse salivary gland proteins and used to select Nbs against the highly immunogenic Tsal proteins by a procedure of phage display and selection for binding onto the recombinant Tsal proteins. One Nb family was identified with the appropriate characteristics for the development of a competitive assay to detect Tsal-specific antibodies raised by the mammalian host when exposed to tsetse fly bites. In this immunoassay, exposure was detected by the inhibition of Nb binding by tsetse fly saliva induced antibodies in plasma. Evaluation of the competitive ELISA test using a set of porcine plasmas revealed an improved accuracy as compared to previously described tests. Moreover, the advantage of this assay is that it does not require adaptation to the sampled host species. We propose the Nb-based competitive ELISA as an additional tool to the indirect ELISA to serologically detect tsetse bite exposure and to monitor the impact of vector control programs and to detect re-invasion of cleared areas by tsetse flies on the African continent. In addition, the concept of using Nbs for the development of competitive antibody detection tests is innovative and broadens the scope of medical diagnostic applications of Nbs.

## Introduction

Control of the tsetse fly vector population represents an important component of the fight against Human and Animal African Trypanosomiasis (HAT and AAT). In addition to the implementation of conventional vector control interventions, alternative strategies [e.g. miniaturized insecticide-treated targets [1], the release of sterile male insects [2,3]] are being deployed on increasingly large scales on the African continent. With the HAT elimination phase in sight [4,5], adequate monitoring of the evolution of tsetse fly densities in areas under tsetse control is a necessity. Here, sero-epidemiological surveys based on tsetse salivary proteins could allow convenient monitoring of tsetse fly exposure on a regular basis and reveal the efficacy of applied and/or ongoing tsetse fly control activities [reviewed in [6]].

Studies using various tsetse fly species have shown that salivary components are immunogenic in mice, rabbits, cattle and humans with the induction of antibodies [7–12]. The secreted saliva proteome (or sialome) of the most studied tsetse species, *Glossina morsitans morsitans*, is predicted to contain more than 200 different protein constituents [13] from which some were described to support the blood feeding physiology [14,15]. Immunoblotting of salivary proteins provided evidence for the immunogenicity of several of the major protein families including endonuclease (Tsal), adenosine deaminase (TSGF), 5’nucleotidase (5’Nuc) and Antigen 5 (Ag5) related proteins [7,8]. In addition, immune screening of a phage salivary gland cDNA expression library resulted in the identification of the immunogenic *G*. *m*. *morsitans* salivary gland proteins *sgp1*, *sgp2* and *sgp3* [16]. A number of potential candidates were proposed as individual exposure biomarkers in the form of recombinant proteins or peptides corresponding to predicted B cell epitopes [17,18]. The TSGF1_18–43_ peptide was shown in West Africa to detect human antibody levels that correlated with the anticipated levels of tsetse exposure [17]. Especially the 43–45 kDa tsetse salivary gland (Tsal) protein family, encoded by 3 *tsal* genes that colocalize to a 10-kb locus in the tsetse fly genome [19], was found to be highly immunogenic with immunoglobulin responses detected in humans living in Uganda [7], Democratic Republic of Congo [10] and Guinea [8]. Corroborating the high immunogenicity, exposure of mice to a single tsetse bite was sufficient to induce detectable levels of anti-Tsal antibodies in plasma. Moreover, murine antibody titers persisted long and remained detectable up to one year after initial challenge [18]. Further exploiting the highly immunogenic nature of the Tsal proteins, rTsal1 was evaluated as antigen in an indirect ELISA test and shown adequate to detect tsetse induced antibody responses in experimentally exposed pigs [18].

With the advent of Nanobody (Nb) technology, the generation of an expression library of monoclonal antigen-binding antibody fragments directed against the tsetse salivary proteome was enabled for protein functional studies. Nbs are moieties of approximately 15 kDa derived from Heavy-chain Antibodies that are present in *Camelidae* [20] and can be selected through phage-display technology and an array of panning methodologies. Nbs are excellent affinity reagents with a small size, high stability, particular epitope range as compared to conventional antibodies, reversible refolding capacity and high solubility in aqueous solutions. Due to these favorable biochemical and biophysical properties, Nbs are increasingly exploited in protein structure/function studies and in the development of medical diagnostic and therapeutic applications (reviewed in ref [21]). Here we report on the selection of a particular Nb family from the anti-tsetse sialome Nb library that is able to mark the presence of anti-Tsal antibodies in plasmas of tsetse fly exposed animals using a competitive ELISA format. Performance of this novel assay is compared with the previously reported indirect antibody detection assay.

## Methods

### Ethics statement

Alpaca immunization and blood collection was performed by the Nanobody Service Facility, VIB. Breeding and experimental work with tsetse flies was approved by the Scientific Institute Public Health department Biosafety and Biotechnology (SBB 219.2007/1410). The experiments, maintenance and care of animals complied with the guidelines of the European Convention for the Protection of Vertebrate Animals used for Experimental and other Scientific Purposes (CETS n° 123).

### Plasmas

Plasma from tsetse fly exposed hyperimmune rabbits was collected in the frame of another study [16]. Mouse plasmas (*n* = 5/group) were available from a previous tsetse fly cross-reactivity study [18], where mice were exposed every 3 days for 6 weeks to 10 flies of either *G*. *p*. *gambiensis*, *G*. *pallidipes* or *G*. *f*. *fuscipes*. Plasma samples were also collected earlier from mice intradermally immunized with decreasing amounts of saliva (5, 2, 1 and 1 μg) harvested from *G*. *m*. *morsitans*, *Stomoxys calcitrans* and *Tabanus yao*. Immune plasmas were collected 10 days after the last exposure. Plasmas obtained from non-exposed mice served as negative controls.

Porcine plasma samples were obtained previously from a total of 10 female pigs exposed to three different *G*. *m*. *morsitans* exposure regimens (high exposure, low exposure and negative control) [18]. From those animals, pre-immune plasmas were collected and samples were collected for 11 successive weeks and after a 2-month period of non-exposure, 2 pigs from the low exposure group were re-challenged by the bites of 10 *G*. *m*. *morsitans* flies followed by weekly plasma collection over a period of 6 weeks.

### Salivary antigens and recombinant proteins

Total *G*. *m*. *morsitans* saliva was obtained as salivary gland outflow from 300 gland pairs incubated for 2h on ice in a sterile physiological salt solution. Saliva was collected as the supernatant after a 2 min. centrifugation at 500 x *g* at 4°C. The saliva protein concentration was determined by the BCA protein assay kit (Pierce Biotechnology) and aliquots for immunization and panning were stored at-80°C.

Salivary gland membrane extracts were prepared from approximately 1200 *G*. *m*. *morsitans* salivary gland pairs from which the saliva outflow was removed. Salivary glands were sonicated in 10 ml phosphate buffered saline (PBS) containing a protease inhibitor mix (Complete, Roche). The supernatant obtained after 30’ centrifugation at 100000 x *g* at 4°C was a salivary gland soluble extract. The salivary gland membrane extract was prepared by resuspending the pellet in 2 ml 2% octyl-β-D-glucopyranoside (in PBS) followed by a 30’ centrifugation at 100000 x *g* at 4°C. The supernatant was collected as the salivary gland membrane extract. The membrane extract was dialysed twice against 250 ml PBS containing 0.05% octyl-β-D-glucopyranoside. Composition was analyzed by protein gel electrophoresis and Coomassie staining. Aliquots for immunization and panning were stored at-80°C.

Recombinant proteins Tsal1 and Tsal2 were purified as described elsewhere from the periplasm of bacterial expression clones using Ni-NTA superflow affinity chromatography (Qiagen) and Superdex 200 gelfiltration chromatography on an Äkta Explorer (GE Healthcare) [22]. Aliquots were stored at −20°C till further use.

### Alpaca immunization and anti-tsetse sialome Nb-library construction

An alpaca was used for immunization with *G*. *m*. *morsitans* saliva and salivary gland membrane extract on contralateral sites of the alpaca body at weekly intervals for six consecutive weeks by injections of 100 μg salivary gland membrane extract and 100 μg saliva in presence of Gerbu adjuvant. Peripheral blood was taken four days after the last injection for the isolation of blood lymphocytes and to assess the antibody response against the salivary antigens. Conventional and heavy chain antibodies were purified from the plasma by protein A and protein G sepharose affinity chromatography. Conventional IgG_1_ antibodies were purified using protein G sepharose, a wash step with 150 mM NaCl 0.58% acetic acid pH 3.5 to remove IgG_3_ and an elution using 100 mM glycine-HCl pH 2.7. Heavy chain IgG_2_ was purified by a negative selection on protein G sepharose and a purification with protein A sepharose followed by a selective elution at pH 3.5. As controls, the same IgG fractions were also purified through the same procedure from an alpaca immunized against a non-related antigen. Reactivity of the purified antibodies was evaluated in an indirect ELISA developed using an in-house rabbit anti-camel polyclonal IgG and a peroxidase-conjugated anti-rabbit IgG (Sigma).

Lymphocytes were isolated from peripheral blood ½ diluted in RPMI1640 using Lymphoprep (Nycomed). RNA was extracted using Trizol reagent and cDNA was prepared from 40 μg total RNA for the construction of the anti-salivary gland immune Nb library. VHH gene fragments ranging from codons from framework region 1 to framework region 4 were amplified by nested PCR. The first PCR round (32 cycles 1’ at 94°C, 1’ at 55°C and 1’ at 72°C) was performed with primers *callI* (5’-GTCCTGGCTGCTCTTCTACAAGG-3) and *callII* (5’-GGTACGTGCTGTTGAACTGTTCC-3’). PCR products were separated on a 2% agarose gel and the 700 bp band corresponding to the amplified VHHs fragments was excised from gel and purified using the QIAquick gel extraction protocol (Qiagen). Using this purified product as template, the 2^nd^ round PCR was performed using the same PCR conditions as mentioned above with primers *A6E* (5’-GATGTGCAGCTGCAGGAGTCTGGRGGAGG-3’) and *pmcf* (5’-CTAGTGCGGCCGCTGAGGAGACGGTGACCTGGGT-3’). Nested PCR products and the pMECS phagmid, derived from pHEN4, were digested with *Pst*I and *Not*I. A total of 17.6 μg of pMECS vector and 18.6 μg of insert were used in the ligation reaction and subsequently transformed with a 1.8 kV pulse into electrocompetent *Escherichia coli* TG1 cells (Immunosource). Electroporated cells were collected in recovery medium (Immunosource) and plated on LB agar plates supplemented with 2% glucose and 100 μg/ml ampicillin. Dilutions were plated to determine the library size and to determine insert frequencies based on a colony PCR (32 cycles 1’ at 94°C, 1’ at 55°C and 1´ at 72°C) using primers *MP57* (5’-TTATGCTTCCGGCTCGTATG-3’) and *GIII* (5’-CCACAGACAGCCCTCATAG-3’). The library was collected in LB-medium supplemented with 20% glycerol, aliquoted and stored at −80°C.

### Selection, purification and HRP-labeling of Tsal-specific Nbs

The anti-tsetse sialome library was expressed on phages and obtained by super-infection of the phagemid-containing TG1 cells with M13K07 helper phages (Invitrogen). Libraries were enriched by four consecutive rounds of *in vitro* selection on a equimolar mix of recombinant Tsal1 and Tsal2 (500 ng/well each, 1 μg in total), immobilized in NUNC MaxiSorp plates (Thermo Scientific). Phages were eluted under alkaline conditions with 100 mM triethylamine (~ pH 11.5), followed by an immediate pH neutralization by 1:1 addition of 1 M Tris pH 7.4 and amplification of eluted phages in *E*. *coli* TG1 cells. Enrichment of the library was assessed by a Tsal1&2-specific phage ELISA using a horseradish peroxidase-conjugated anti-M13 antibody (Amersham Biosciences). Individual colonies of transfected TG1 cells from the third panning round were picked to evaluate the expression of Tsal-specific Nbs upon induction with 1 mM isopropyl-β-d-thiogalactopyranoside (IPTG). Binding was evaluated onto recombinant Tsal1&2 (500 ng/well each) and native Tsal proteins in *G*. *m*. *morsitans* saliva (1 μg/well). Detection was with a peroxidase-conjugated mouse anti-HA IgG1 (HA.11 Clone 16B12, BioLegend) and with TMB substrate (3,3′,5,5′-Tetramethylbenzidine, Sigma). Reactions were stopped by the 1:2 addition of 1 N H_2_SO_4_ and optical densities (O.D.) were measured using a Multiskan Ascent plate reader (Thermo) at a 450 nm wavelength.

Tsal-specific clones were subjected to sequence analysis (Genetic Service Facility, VIB). Translated sequences were aligned using the CLUSTALW program and imported into GeneDoc (http://www.psc.edu/biomed/genedoc). Aligned sequences were imported in Mega 5 and consensus maximum likelihood trees were built using the standard settings and 500 bootstrap replications.

Clones of interest were used for Nb purification directly from TG1 cells. Expression was induced overnight at 28°C with 1 mM IPTG, Nbs were purified from periplasmic bacterial extracts with Ni-NTA columns (Qiagen) using 0.5 M imidazole/PBS as elution buffer followed by purification by size exclusion chromatography on a Superdex 75 10/300 GL column (GE Healtcare) with phosphate buffered saline (PBS) as running buffer. Protein concentrations were determined by the optical density at 280 nm and the individual theoretical extinction coefficients calculated using the ProtParam webtool. Nbs were aliquoted and stored at-20°C until further use.

Nanobodies were labeled by use of EZ link Plus Activated Peroxidase (Thermo Scientific). Conjugation reactions were carried out for 1h at room temperature at pH 7.2, followed by quenching the reaction and purification by size exclusion chromatography on a Superdex 75 10/300 GL column. Conjugates were stored at 4°C.

### Nanobody binding kinetics and affinity determination

To evaluate the binding kinetics of selected Nbs for the native Tsal proteins, 4000 resonance units (RU) corresponding to approximately 4 ng/mm² of *G*. *m*. *morsitans* saliva was coupled in 10 mM sodium acetate pH 4.5 onto a CM5 chip (BIAcore) using EDC and NHS as cross-linking agents and ethanolamine to block free esters. The individual purified Nb analytes were tested in concentrations ranging from 500 to 0.976 nM on a BIAcore T200 apparatus. Contact time was for 180 s, dissociation for 600 s at a 10 μl/min flow rate. Chip regeneration was achieved with 2 pulses of 10 s with 100 mM glycine-HCl pH 2.2 at a 30 μl/min flow rate. Sensograms were fitted for a Langmuir 1:1 binding model using the BIAcore T200 evaluation software version 1.0, resulting in association and dissociation constants (k_a_ and k_d_) as output from which affinity (K_D_) values were calculated. χ^2^ values and residuals were analyzed for accuracy of the fitting. Epitope mapping was performed on the CM5-*G*. *m*. *morsitans* saliva sensor chip by evaluating cumulative or competitive binding of two-by-two Nb combinations at a 500 nM concentration.

Steady state affinities were also evaluated by ELISA where Nbs were applied to *G*. *m*. *morsitans* saliva coated wells and detected using the HRP-conjugated anti-HA Tag antibody and TMB as substrate. Responses to the antigen (O.D._450nm_ values) were fitted to a one-site binding non-linear regression model in the GraphPad Prism 5.02 software package with B_max_ and K_D_ values as output parameters.

### Serological analyses

Polystyrene 96 well plates (NUNC MaxiSorp™ Surface, Thermo Scientific) were coated for 1h at ambient temperature with 250 ng *G*. *m*. *morsitans* saliva in 0.1 M NaHCO_3_ (pH 8.3) in each well. Plates were overcoated for 1 h with 10% fetal bovine serum (FBS) at ambient temperature. Diluted or undiluted plasma samples were applied for 1 h. Next, plates were incubated for 1h with appropriate dilutions (1:160 or 1:320) of the HRP-conjugated Nbs in PBS. Detection was with ABTS substrate (KPL) and optical densities were measured using the Multiskan Ascent plate reader at a 405 nm wavelength.

### Data analysis

Competitive ELISA results were expressed as endpoint O.D._405nm_ values after 30 min incubation (within the semi-linear phase of substrate conversion). Percentage inhibition was calculated from the rate of substrate conversion (ΔO.D._405nm_/min) relative to the activity recorded in wells with control plasmas (100% activity). Inhibition of Nb binding as evaluated by two-way analysis of variance and pairwise comparisons were performed using a Bonferroni post-test. Cross-reactivity of immune responses induced by different *Glossina* species and other hematophagous insects was analysed using a one-way analysis of variance and Tukey’s multiple comparisons technique. Graphs were prepared using the GraphPad Prism 5.02 software package. Data represented in the graphs are the means and the 95% confidence intervals (CI).

Competitive ELISA results for the two selected Nb candidates (Nb-Tsal-05 and Nb-Tsal-11) were compared by the non-parametric Spearman correlation test using GraphPad Prism 5.02 with Spearman correlation coefficient (*r*) as output. Performance of the assays was assessed by receiver operating characteristic (ROC) curve analysis of the endpoint O.D._405nm_ values of non-exposed animals (21 control plasmas, including 9 pre-immune and 12 repeated samplings of the control pig) and exposed animals 3 weeks after the initial exposure (90 plasmas obtained by repeated sampling of the 9 exposed pigs). The area under the ROC curve (AUC) was used as a global index of diagnostic accuracy. To compare the assays with the previously described *G*. *m*. *morsitans* and rTsal1 based indirect ELISA [18], we have calculated sensitivity, specificity, positive and negative predictive values and accuracy on the basis of the same set of 111 porcine plasmas used to make the ROC curve analysis. Positive samples were identified based on cut-off values calculated using a Student *t*-distribution according to Frey *et al*. [23]. For the indirect ELISA, the upper prediction limit was determined with following formula using a 95% confidence level:

$$Cut-off=\bar{X}+\left(SD\times t\sqrt{1+(1/n)}\right)$$

For the competitive ELISA, the lower prediction limit was used as cut-off, calculated as follows:

$$Cut-off=\bar{X}-\left(SD\times t\sqrt{1+(1/n)}\right)$$

## Results

### Generation of an anti-tsetse sialome Nb library and selection of Tsal-specific Nbs

**Figure 1:**
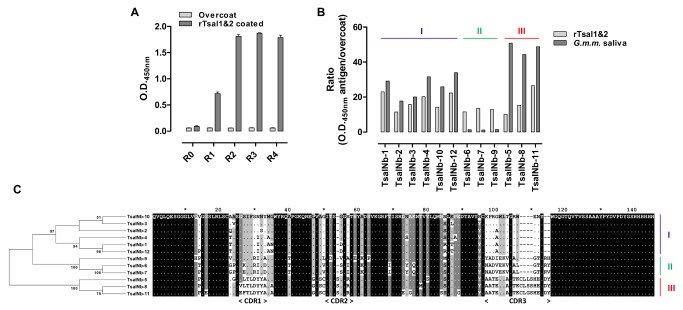
Selection of Tsal-specific Nbs from the anti-tsetse sialome Nb library. (**A**) Enrichment of Tsal-specific phages during the different panning rounds as determined by phage ELISA. (**B**) ELISA to detect Tsal-specific Nb-binding activity in periplasmic extracts of individual clones. Binding was evaluated onto the immobilized recombinant Tsal1 and Tsal2 and onto native Tsal in total *G*. *m*. *morsitans* saliva. (**C**) Amino acid sequences of the 12 identified TsalNb clones with the corresponding maximum likelihood consensus tree. Three Nb families were identified based on sequence variations in CDR-1 and CDR-3 (TsalNb families I, II and III) with different binding properties as observed in the periplasmic extract ELISA. Dots in the alignment represent conserved amino acid positions.

**Figure 2:**
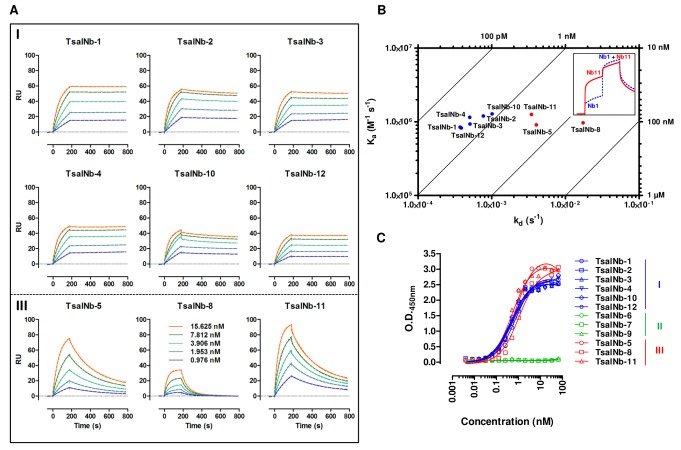
Functional characterization of the Tsal-specific Nbs. (**A**) Surface plasmon resonance experimental (SPR) sensograms (solid line) and the fitted data (dotted line) obtained for different TsalNb analyte concentrations (15.125–0.976 nM) with 4000 RU *G*. *m*. *morsitans* saliva immobilized on a CM5 chip. Upper panel are sensograms for TsalNbs of family I, lower panel those of family III. (**B**) Dot plot representation with isoaffinity lines of the individual k_a_, k_d_ and K_D_ affinity constants (see Table 1) of the different TsalNbs as determined by SPR. Blue symbols represent TsalNbs of family I, red symbols represent TsalNbs of family III. Inset: epitope mapping of TsalNb-1 (family I) and TsalNb-11 (family III) in SPR, revealing cumulative binding reminiscent of a different epitope recognition (**C**) Binding studies in ELISA with purified TsalNbs-1–12 (TsalNb families I, II and III shown in blue, green and red respectively) onto solid phase immobilized *G*. *m*. *morsitans* saliva detected using an HRP-conjugated anti-HA Tag antibody.

Immunization of an alpaca against soluble saliva antigens and the salivary gland membrane proteins resulted in strong antibody responses, both in the conventional (IgG_1_) and heavy chain antibody (IgG_2_) isotypes as determined by an indirect ELISA. A Nb library was constructed from the peripheral blood lymphocytes of the immunized alpaca by a VHH-specific nested RT-PCR and by ligation of the obtained inserts into the phagemid vector pMECS. The generated library had a size of 1.3 10^7^ individual transformants for which it was estimated that 80% harboured the vector with an insert of the correct size as determined by colony PCR. Four consecutive panning rounds were performed on solid phase immobilized recombinant Tsal1 and Tsal2 with a clear enrichment recorded by phage ELISA (Fig. 1A). A total of 12 different clones (TsalNb1–12) belonging to three Nb families (I—III) were identified based on sequence variations in the complementarity determining regions CDR-1 and CDR-3 (Fig. 1C). Periplasmic extract ELISA revealed variations in binding profiles of the different Nb families (Fig. 1B), with family II displaying a nearly exclusive preference for the recombinant form of the Tsal proteins and Nb family III the highest antigen/overcoat O.D. ratio in favour of the native Tsal proteins in *G*. *m*. *morsitans* saliva. Surface plasmon resonance (SPR) analysis was used to obtain kinetic data of binding of family I and III TsalNbs onto the native Tsal proteins in *G*. *m*. *morsitans* saliva (Fig. 2A&B). Consistent with the ELISA results, family II TsalNbs were achieving very low binding responses in SPR with affinities in the micromolar range. Without emphasizing on the absolute values calculated for the affinity and kinetic rate constants for the interaction between the Nb analytes and the heterogenous saliva ligand, family I TsalNbs consistently dissociated slower from their antigenic target (with smaller k_d_ dissociation constants) as compared to family III TsalNbs (Table 1, Fig. 2A&B). Results obtained by SPR kinetic analyses (Table 1) and binding studies performed by ELISA (Fig. 2C) are compatible with affinities in the high picomolar range for the family I TsalNbs and in the low nanomolar range for the family III TsalNbs. The highest binding responses in SPR and ELISA were obtained with TsalNb-5 and TsalNb-11 of TsalNb family III. Epitope mapping by SPR revealed cumulative binding of TsalNb1 and TsalNb11, demonstrating that Nbs of families I and III bind to distinct epitopes (Fig. 2B, inset).

**Table 1:** Characteristics of the selected anti-Tsal Nbs.

Nb family	ID	k_a_ (M^-1^s^-1^)	k_d_ (s^-1^)	K_D_ (M)
I	TsalNb-1	8,5E+05	3,7E-04	4,4E-10
	TsalNb-2	1,2E+06	7,7E-04	6,4E-10
	TsalNb-3	9,3E+05	5,1E-04	5,4E-10
	TsalNb-4	1,1E+06	5,0E-04	4,4E-10
	TsalNb-10	1,3E+06	1,0E-03	7,9E-10
	TsalNb-12	8,3E+05	3,9E-04	4,7E-10
III	TsalNb-5	9,1E+05	4,0E-03	4,4E-09
	TsalNb-8	9,7E+05	1,7E-02	1,8E-08
	TsalNb-11	1,3E+06	3,5E-03	2,7E-09

The affinity and kinetic rate constants determined by surface plasmon resonance on a CM5-*G*. *m*. *morsitans* saliva chip with the TsalNbs of families I and III.

### Development of a Nb-based competitive immunoassay

**Figure 3:**
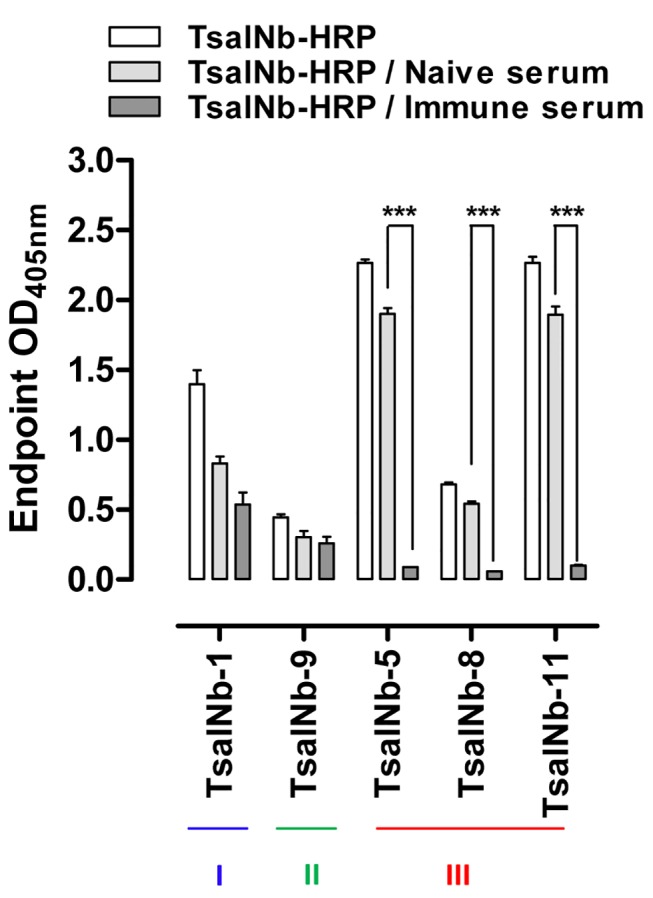
Competitive inhibition of TsalNb binding by immune plasma. Representatives of the identified anti-Tsal Nb families (TsalNb-1, TsalNb-9 and TsalNb-5,8&11) were covalently conjugated to HRP and evaluated for Tsal-binding (O.D._405nm_) following incubation of the coated *G*. *m*. *morsitans* saliva with tsetse exposed immune or naive rabbit plasma. Significance levels based on two-way analysis of variance are indicated in the graphs (*** p<0.001).

Representatives of the 3 Tsal Nb families (TsalNb-1, TsalNb-9 and TsalNb-5,8&11) were chemically conjugated to horseradish peroxidase (HRP), purified by size exclusion chromatography to remove unlabeled Nb and used as detection moieties in the *G*. *m*. *morsitans* saliva based ELISA assay. Evaluation of the inhibition of Nb binding by immune plasma from tsetse fly exposed rabbits, provided strong statistical support for a significant competition with TsalNb-5,8&11 (*p* < 0.001, Fig. 3), representatives of TsalNb family III. To confirm the potential of TsalNb family III for the development of a competitive anti-Tsal antibody detection assay, the two different Nbs with the highest naive/immune O.D. ratios—TsalNb-5 and TsalNb-11—were HRP conjugated in an independent labeling reaction and purified (S1A Fig.), titrated to determine the optimal working Nb-HRP dilution (S1B Fig.) and evaluated in a competitive assay using various rabbit serum concentrations (S1C Fig.). A strong inhibition of Nb binding by the immune serum was observed for the two Nb-HRP conjugates.

### Cross-reactivity profile of the Nb-based competitive immunoassay

**Figure 4:**
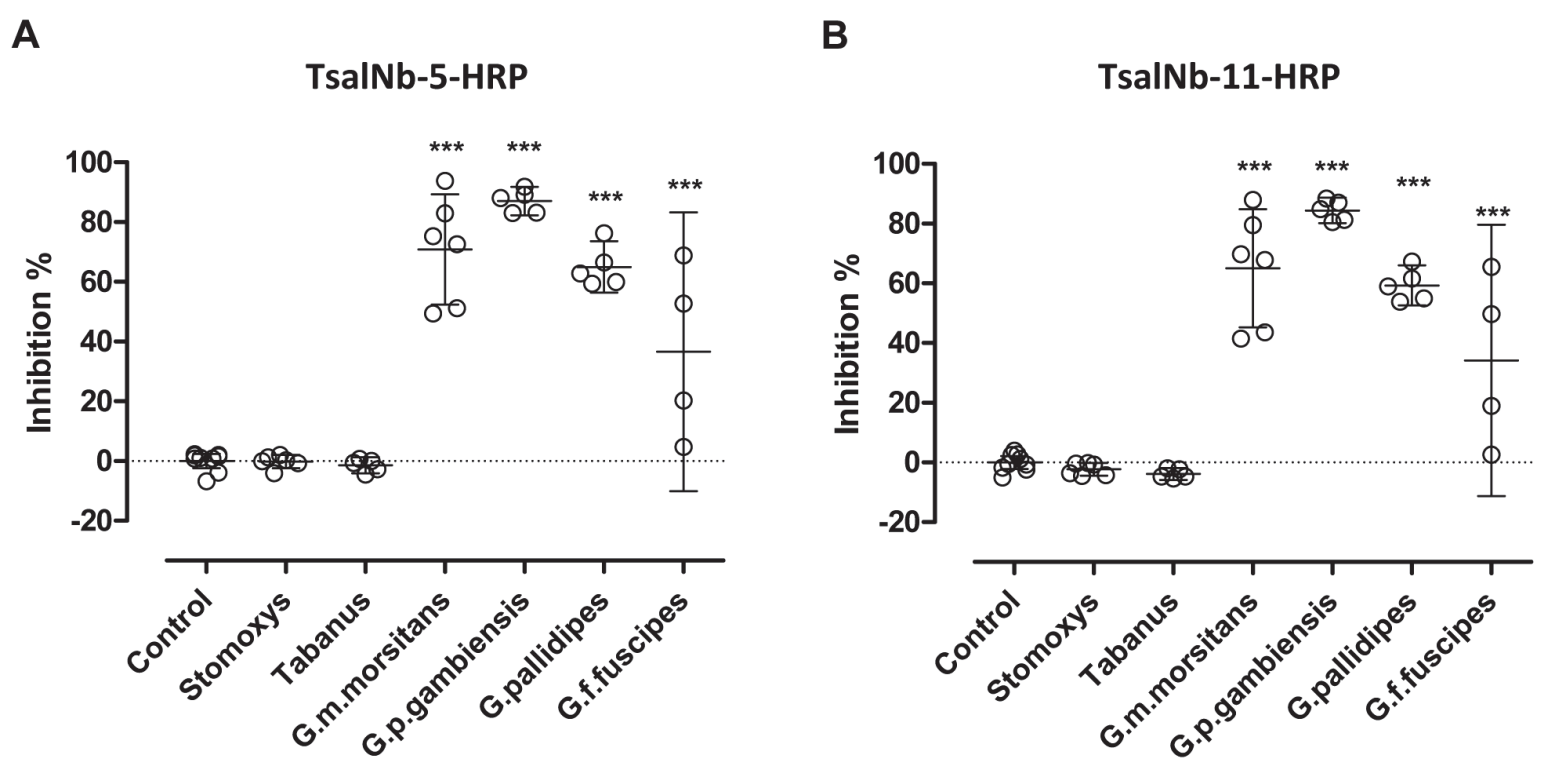
Cross-reactivity profile of the Nb-based competitive immunoassay. Plasmas of control mice and mice repeatedly exposed to the bites of various tsetse species (*G*. *palpalis gambiensis*, *G*. *pallidipes* and *G*.*fuscipes fuscipes*) and mice intradermally immunized with harvested *G*. *m*. *morsitans*, *Stomoxys calcitrans* and *Tabanus yao* saliva were analysed for anti-Tsal antibody responses in the competitive ELISA using TsalNb-5-HRP (**A**) and TsalNb-11-HRP (**B**) as detection reagents. The mean endpoint O.D._405nm_ of controls were 2.260 and 2.354 following detection with respectively TsalNb-5-HRP and TsalNb-11-HRP. Presented data are the mean percentage inhibition relative to the controls with the 95% CI. Significance levels based on one-way analysis of variance are indicated in the graphs (*** p<0.001).

In order to evaluate the cross-reactivity with other tsetse fly species, mouse plasmas immunized against the saliva of various *Glossina* species (*G*. *p*. *gambiensis*, *G*. *pallidipes* and *G*. *f*. *fuscipes)* were tested in the competitive immunoassay using TsalNb-5-HRP and TsalNb-11-HRP. A marked cross-reactivity of the anti-saliva antibodies elicited by the three *Glossina* species (*G*. *p*. *gambiensis* and *G*. *pallidipes*, *G*. *f*. *fuscipes*; *p* < 0.001) was detected (Fig. 4A&B). Only one *G*. *f*. *fuscipes* exposed animal was scored as false negative on the basis of a cut-off determined from the control samples. No false positive reactions were observed in any of the control animals.

In order to evaluate cross-reactivity with other hematophagous insects, mouse plasmas exposed to the saliva of stable flies (*Stomoxys calcitrans*) or horse flies (*Tabanus yao*) and samples from control mice and mice that were immunized with *G*. *m*. *morsitans* saliva following the same immunization protocol were tested. Immunization with *Stomoxys* and *Tabanus* saliva did not result in antibodies that false positively reacted in the competitive assay indicating that the assay is tsetse fly specific (Fig. 4A&B).

### Evaluation of the Nb-based competitive immunoassay in pigs exposed to *G*. *morsitans* bites

**Figure 5:**
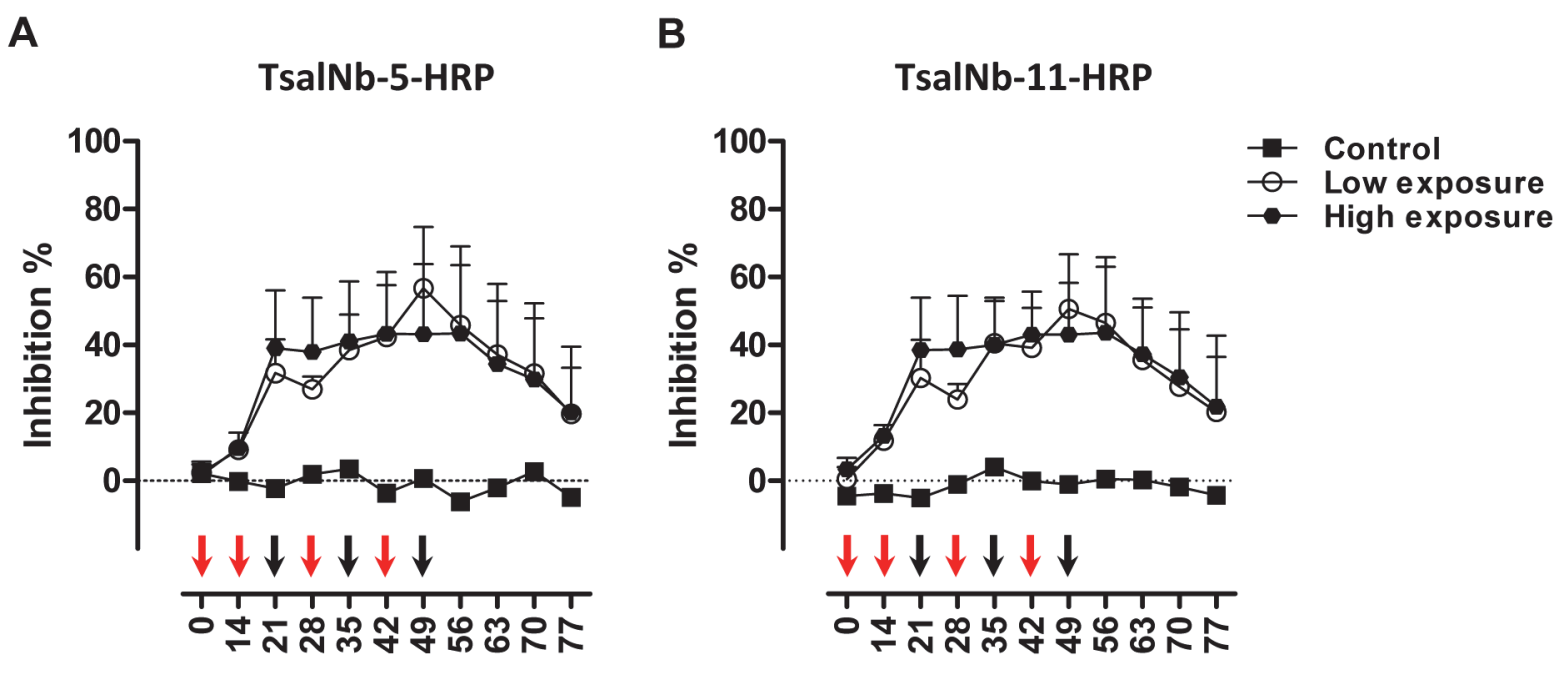
Detection of humoral responses in experimentally exposed pigs. Follow-up of the anti-Tsal antibody responses in pigs exposed to 2 different tsetse fly biting intensities [two-weekly exposure for 6 weeks to 3 flies (low exposure group, n = 4) or weekly exposure for 7 weeks to 30 flies (high exposure group, n = 5)] in the competitive ELISA using TsalNb-5-HRP (**A**) and TsalNb-11-HRP (**B**) as detection reagents. Samples from one non-exposed animal were available as negative controls. The mean endpoint O.D._405nm_ of controls were 2.315 and 2.240 following detection with respectively TsalNb-5-HRP and TsalNb-11-HRP. Presented data are the mean percentage inhibition relative to the controls with the 95% CI obtained for undiluted plasmas.

**Figure 6:**
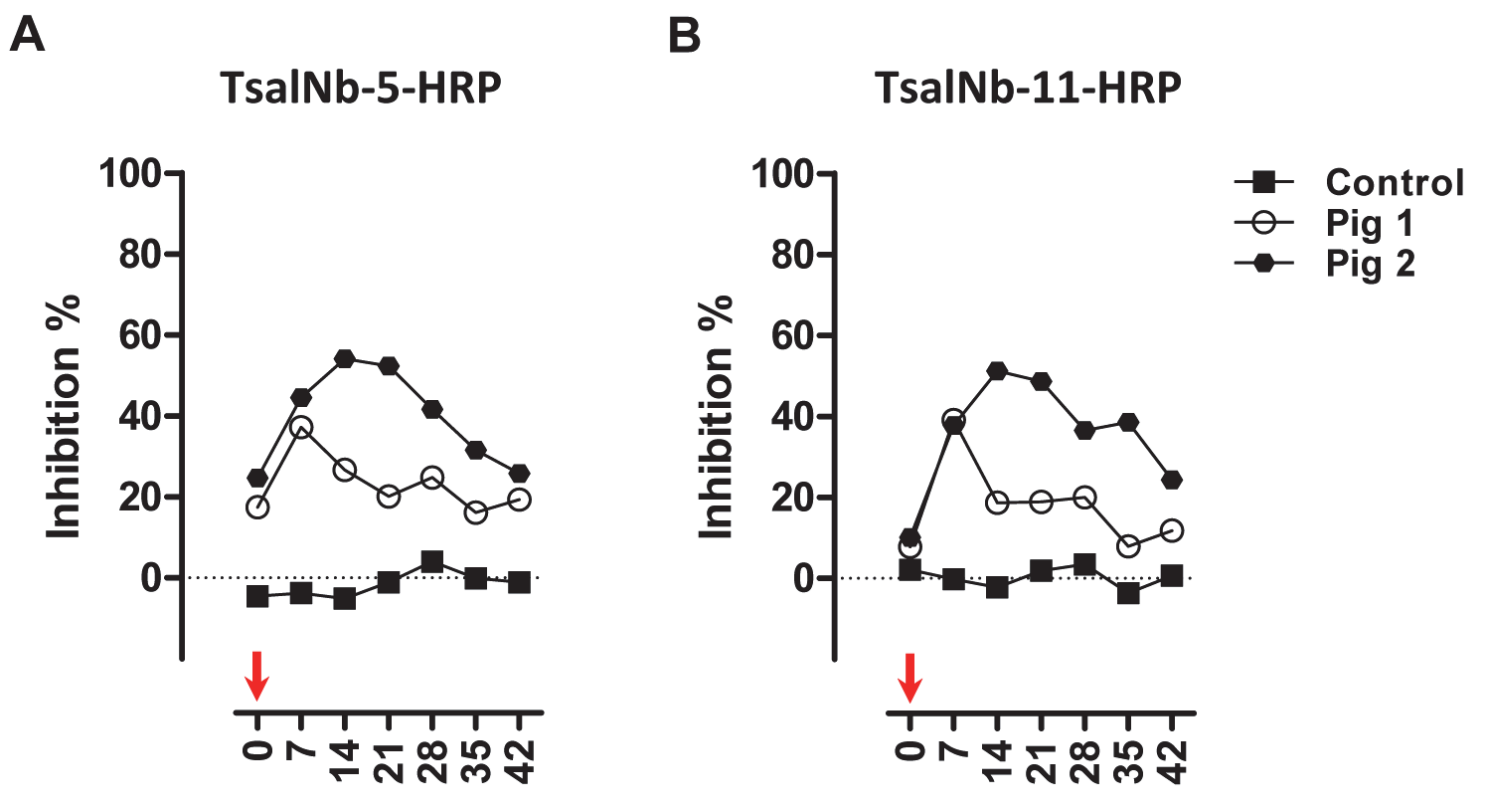
Detection of boosting in pigs after a period of non-exposure. Evolution of the anti-Tsal antibody responses in the competitive ELISA using TsalNb-5-HRP (**A**) and TsalNb-11-HRP (**B**) was evaluated in pigs (*n* = 2) originally exposed two-weekly for 6 weeks to the bites of 3 flies (= low exposure group) and re-exposed to the bites of 10 flies (indicated by an arrow) after a 2-month period of non-exposure. One non-exposed animal sampled during the priming experiment (Fig. 5) served as a negative control. The mean endpoint O.D._405nm_ of controls were 2.235 and 2.291 following detection with respectively TsalNb-5-HRP and TsalNb-11-HRP. Presented data are the percentages inhibition relative to the controls obtained for the plasmas of the individual pigs.

Plasma samples were previously collected from pigs that were experimentally exposed to a low or a high tsetse fly bite regimen. Titration studies using plasma collected at the peak of anti-Tsal1 and anti-*G*. *m*. *morsitans* saliva antibody responses as determined earlier [18], indicated that best competition was obtained with undiluted porcine plasma (*p* < 0.05; S2 Fig.). Evaluation of the anti-Tsal responses over time with the competitive assay revealed similar kinetics as determined earlier with the *G*. *m*. *morsitans* saliva and rTsal1 based indirect ELISA. A steady decline in antibody titers was observed upon cessation of tsetse exposure (Fig. 5A&B). However, unlike the indirect ELISA, competitive ELISA test results did not correlate significantly with the intensity of tsetse exposure. Boosting of two immunized pigs from the low exposure group after a 2-month non-exposure period by the bites of 10 flies resulted in elevated anti-saliva antibody titers that were detectable in the competitive immunoassay (Fig. 6A&B). A good correlation between the TsalNb-5-HRP and TsalNb-11-HRP based tests was recorded with a Spearman correlation coefficient *r* of 0.95 (S3A Fig.). Area under the ROC curve (AUC = 0.99, S3B Fig.) for the TsalNb-5-HRP and TsalNb-11-HRP immunoassays were very high and exceeded AUCs that we reported previously for the porcine indirect ELISA using respectively *G*. *m*. *morsitans* saliva and rTsal1 as coating antigens (AUC = 0.96 and 0.83, [18]). Given the absence of a gold standard, we made a comparison of the new competitive test with the previously described indirect ELISA tests in terms of sensitivity, specificity, positive and negative predictive values (PPV and NPV) and accuracy determined for the available set of experimentally exposed porcine plasmas (*n*_exposed_ *= 90*, *n*_non-exposed_ = 21, Table 2). These analyses showed that all assays under their specific test conditions had very good specificities (> 95%) and PPVs (> 98%). However, the competitive assay appeared to result in less false negative diagnoses, resulting in higher NPVs and hence increased sensitivity (> 95%) and accuracy (> 95%).

**Table 2:** Comparison of the test characteristics of the novel competitive ELISA and the previously described indirect immunoassays with the available set of porcine plasmas.

	Indirect ELISA		Competitive ELISA	
	*G*.*m*.*m*. saliva	rTsal1	TsalNb-8-HRP	TsalNb-11-HRP
*n*_exposed_	90	90	90	90
*n*_non-exposed_	21	21	21	21
Cut-off O.D. 95% CL	0,202	0,212	2,145	2,066
TP	79	51	87	86
TN	20	20	20	20
FP	1	1	1	1
FN	11	39	3	4
Sensitivity	87,8% (79/90)	56,7% (51/90)	96,7% (87/90)	95,6% (86/90)
Specificity	95,2% (20/21)	95,2% (20/21)	95,2% (20/21)	95,2% (20/21)
PPV	98,8% (79/80)	98,1% (51/52)	98,9% (87/88)	98,8% (86/87)
NPV	64,5% (20/31)	33,9% (20/59)	87,0% (20/23)	83,3% (20/24)
Accuracy	89,2% (99/111)	64,0% (71/111)	96,4% (107/111)	95,5% (106/111)

Respective ELISA results for a set of 90 tsetse exposed (10 repeated samplings of the 9 exposed pigs starting three weeks after the initial exposure) and 21 non-exposed porcine plasmas (9 pre-immune and 12 repeated samplings of the control pig) were interpreted based on the calculated cut-off values (see methods section). TP true positive, TN true negative, FP false positive, FN false negative, PPV positive predictive value, NPV negative predictive value.

## Discussion

The concept of exploiting salivary components as specific biomarkers of exposure of vertebrate host to bites of medically important arthropods has been proposed for a number of species of sand flies [24–26], triatomine bugs [27,28], mosquitoes [29–31] and also tsetse flies [7,8,10–12,17,18]. The main added value of such approach is that relatively simple serological tests could indicate actual exposure to the bites of disease vectors without the requirement of conducting strenuous entomological surveys. Beside serving as a risk indicator for infection with a vector-transmitted pathogen, this approach might reveal low levels of exposure that are undetectable by entomological trappings [32] and allow the monitoring of the efficacy of control interventions [24,30,33,34]. This saliva biomarker approach can be performed at the population-level or can be limited to individual serological follow-up of selected animals or sentinel animals.

In this study we have improved and simplified the detection of tsetse fly induced antibody responses against the Tsal protein family. This was realized by converting the indirect ELISA for Tsal antibody detection that we described previously [18] into a competitive assay format using anti-Tsal nanobodies (Nbs). For this, Tsal-specific Nbs were selected from an anti-*G*. *m*. *morsitans* sialome Nb-library using the recombinant versions of the Tsal proteins that we previously described to exert high affinity nucleic acid binding activity [22]. A total of three Nb families were identified from the anti-sialome Nb library, including two (family I and III) that reacted with different epitopes on the native Tsal proteins. Both Nb families did not react with the rTsal1 protein that was purified under denaturing conditions and that we previously characterized as coating antigen in the indirect ELISA [18], suggesting that these Nbs recognize conformational epitopes. Two Nbs of family III (TsalNb-5 and TsalNb-11), conjugated to HRP, displayed the best binding characteristics and achieved the best results in a competitive assay that was first tested using plasma of tsetse saliva hyperimmune rabbits. Indeed, these two particular Nb candidates achieved highest maximal binding responses in the ELISA and SPR studies, displayed moderate dissociation constants and were efficiently displaced by the polyclonal host antibodies in a competitive assay. The absence of efficient competition with family I TsalNbs could result from a targeted epitope that might not overlap with those of polyclonal antibodies induced by tsetse fly exposure and/or by the very slow dissociation rate that might hamper efficient Nb-HRP displacement. Following the pilot experiments with rabbit hyperimmune plasma, TsalNb-5-HRP and TsalNb-11-HRP were further evaluated with a panel of available murine and porcine plasmas of tsetse exposed animals.

Analysis of mouse plasmas exposed to different tsetse fly species revealed a broad range of detection of the competitive assay, including exposure to *G*. *f*. *fuscipes*. This confirmed our previous observation that the Tsal antigens are sufficiently conserved in the saliva of several *Glossina* species to allow multi-species detection of exposure [18], which was achieved with this new competitive immunoassay. Exposure of mice to the saliva of other hematophagous insects (*Tabanus* and *Stomoxys* sp.) that are known to be abundant in areas where tsetse flies are present, revealed that the competitive assay does not cross-react with those insect species. This feature has also been documented for the *G*. *m*. *morsitans* saliva and rTsal-based indirect ELISA tests [18]. Observations in cattle exposed to *Stomoxys* and *Tabanus* bites revealed that also *G*. *m*. *submorsitans* saliva shares this feature of specificity, unlike *Glossina tachinoides* and *G*. *palpalis gambiensis* saliva [12]. These observations further supported the concept that the Tsal antigens in *G*. *morsitans* saliva are suitable antigenic targets for specific pan-tsetse species exposure detection. Moreover, the detection of responses against a specific diagnostic epitope on the Tsal proteins in the competitive assay strongly reduces the possibility of unspecific reactions, a principle that is also aimed at by immunoassays that use synthetic peptides [17]. The use of total saliva as crude antigen in this assay might be a drawback for two main reasons: (*i*) the production and (*ii*) possible issues with specificity. Given that *G*. *m*. *morsitans* colonies exist at various locations in the world and that the harvesting of saliva can be relatively straightforward and with high yields, this is not necessarily a problem. Production of folded, biologically active Tsal proteins in the periplasm of *E*. *coli* is possible, but yields are very low due to cytotoxicity that is probably linked to the DNA-binding/endonuclease activity of the properly folded Tsal proteins [22]. Other strategies for the efficient production of these proteins, e.g. in insect cells, could be envisaged. Related to the issues of specificity, the detection moiety (a Nb directed against a single diagnostic epitope) circumvents problems of specificity encountered with crude antigens. Indeed, epitopes are presented in the natural conformation and displacement of high affinity Nbs disfavors detection of low affinity cross-reactive antibodies. An advantage of using total saliva is that it could also serve as an antigen in competitive ELISAs using nanobodies against other antigenic targets, enabling a multi-epitope immunoprofiling. This competitive assay has the additional advantage that given the particular assay type, no modifications are required to detect antibodies across host species. Indeed, given particular issues with porcine IgG detection, *E*. *coli* soluble extract had to be added to the sample diluent to increase assay specificity of the rTsal1 and *G*. *m*. *morsitans* saliva based indirect ELISAs [18]. In combination with the prescribed 1:1600 plasma dilution, these required stringent assay conditions for the indirect antibody detection test ensured very good specificities and positive predictive values, exceeding 95% and 98% respectively, but resulting in a reduced negative predictive value and sensitivity. The competitive ELISA did not require this sample treatment and dilution, enhancing the detection of antibodies in pigs exposed to the low exposure regimen. Although the competitive test was unable to differentiate the responses against the one particular diagnostic epitope induced by the low and high exposure regimen, a steady decline in antibody titers could be detected upon cessation of tsetse exposure. A comparison of the competitive assay performance to the previously described assays using an available panel of 21 exposed and 90 non-exposed porcine plasmas revealed less false negative diagnoses and an improved negative predictive value (83–87%), sensitivity (> 95%) and accuracy of the test (> 95%). Estimation of the real test characteristics will require evaluation in the field on a larger scale.

The general diagnostic usefulness of competitive ELISAs is well established and is exemplified by the commercialization of various antibody detection tests for e.g. Rift Valley fever, foot-and-mouth disease, West Nile virus and avian influenza infections that make use of conventional monoclonal antibody technology. In this study we have broadened the scope of application of Nbs by illustrating that they can be used in competitive antibody detection tests. The ease of selecting suitable diagnostic Nbs through phage display and panning provides a promising alternative to monoclonal antibody technology in diagnostic test development. There are already a number of examples of the use of Nbs for the specific detection of components in complex mixtures. For instance, Nbs have been selected with the appropriate characteristics to evaluate the presence of a diagnostic glycoprotein of *Taenia solium* cysticerca in porcine serum [35], to quantify prostate-specific antigen in spiked human serum [36] and to detect the fungal aflatoxin in organic food extracts [37]. However, Nbs had not yet been used for the detection of antibodies. The reason for this is that it is generally assumed that Nbs have different epitope specificities than conventional antibodies given that the paratope of a conventional antibody is constituted by parts of the heavy and light antibody chain which significantly differs from a Nb paratope on a single domain (VHH). Here, we have illustrated that competition can occur between Nbs and conventional antibodies for epitope binding which enables the selection of diagnostic Nb—antigen pairs for antibody detection. Given the possibility of constructing anti-proteome and anti-infectome libraries [38–41] and the ease of selecting monoclonal Nbs by phage display, this paves the way to identifying diagnostic Nbs for a range of antibody detection tests. This approach represents a new biological screening method to identify specific epitopes for the development of serological tests with increased specificity. In the case of detecting tsetse exposure, additional Nb candidates could be selected targeting other salivary biomarkers from the anti-tsetse sialome Nb library to allow multi-target immunoprofiling of hosts.

Collectively, the prominent induction of anti-Tsal antibodies upon tsetse fly exposure has been exploited to develop a novel competitive test using Nb technology which remains to be further evaluated under field conditions. Based on testing of an available set of murine and porcine plasmas, the test characteristics of this immunoassay exceeded those of existing assays and displayed a broad tsetse species range. In addition, the particular assay format implies that no host-specific detection is required, with the possibility of streamlining anti-Tsal antibody detection in a range of hosts living in the tsetse fly belt to provide a measure for tsetse prevalence, the impact of an intervention strategy or the efficacy of a protecting barrier or to detect re-invasion of previously cleared areas.

## Supporting Information

S1 Fig Nanobody labeling and primary competitive ELISA tests. (**A**) Size exclusion chromatography to exclude unlabeled Nbs from the TsalNb-5 (solid line) and TsalNb-11 (dashed line) HRP-conjugation reactions. (**B**) Titration of TsalNb-5-HRP and TsalNb-11-HRP and binding (endpoint O.D._405nm_) onto *G*. *m*. *morsitans* saliva coated and blanc coated wells. TsalNb-HRP working dilutions for subsequent tests were chosen at the start of the declining phase of the Nb-HRP binding curve (arrows). (**C**) Inhibition of TsalNb-HRP binding following incubation of the *G*. *m*. *morsitans* saliva coated wells with several dilutions of tsetse-exposed immune or naive rabbit serum.
(TIF)

S2 Fig Primary competitive ELISA tests with porcine plasma samples. Porcine plasmas of control animals and animals exposed to a low or high tsetse challenge regimen sampled at the peak of anti-Tsal antibody responses were serially diluted and tested in the competitive ELISA. Presented data are the percentage inhibition with the means and 95% CI. Significance levels based on two-way analysis of variance are indicated in the graphs (*p<0.05).
(TIF)

S3 Fig Repeatability and specificity/sensitivity analysis of the competitive immunoassay in pigs. (**A**) Scatter plot analysis of the anti-Tsal antibody responses (O.D._405nm_) detected in undiluted porcine plasma samples (*n* = 90) with the competitive immunoassay using TsalNb-5-HRP and TsalNb-11-HRP for detection. Overall test results with the two Nb-HRP detection moieties were compared using the non-parametric Spearman correlation test with the correlation coefficient *r* as output. (**B**) Sensitivity and specificity of the competitive assays were assessed by receiver operating characteristic (ROC) curve analysis of the O.D._405nm_ values of exposed (*n* = 90) and non-exposed mice (*n* = 21). The area under the ROC curves (AUC) for detection with TsalNb-5-HRP (solid line) and TsalNb-11-HRP (dotted line) are shown as a measure for the individual test performances.
(TIF)
