# Supplementary figures and images for: Description of a Nanobody-based Competitive Immunoassay to Detect Tsetse Fly Exposure
Source: PLoS Negl Trop Dis. 2015 Feb 6;9(2):e0003456. doi: 10.1371/journal.pntd.0003456 (PMC4320081; doi:10.1371/journal.pntd.0003456)

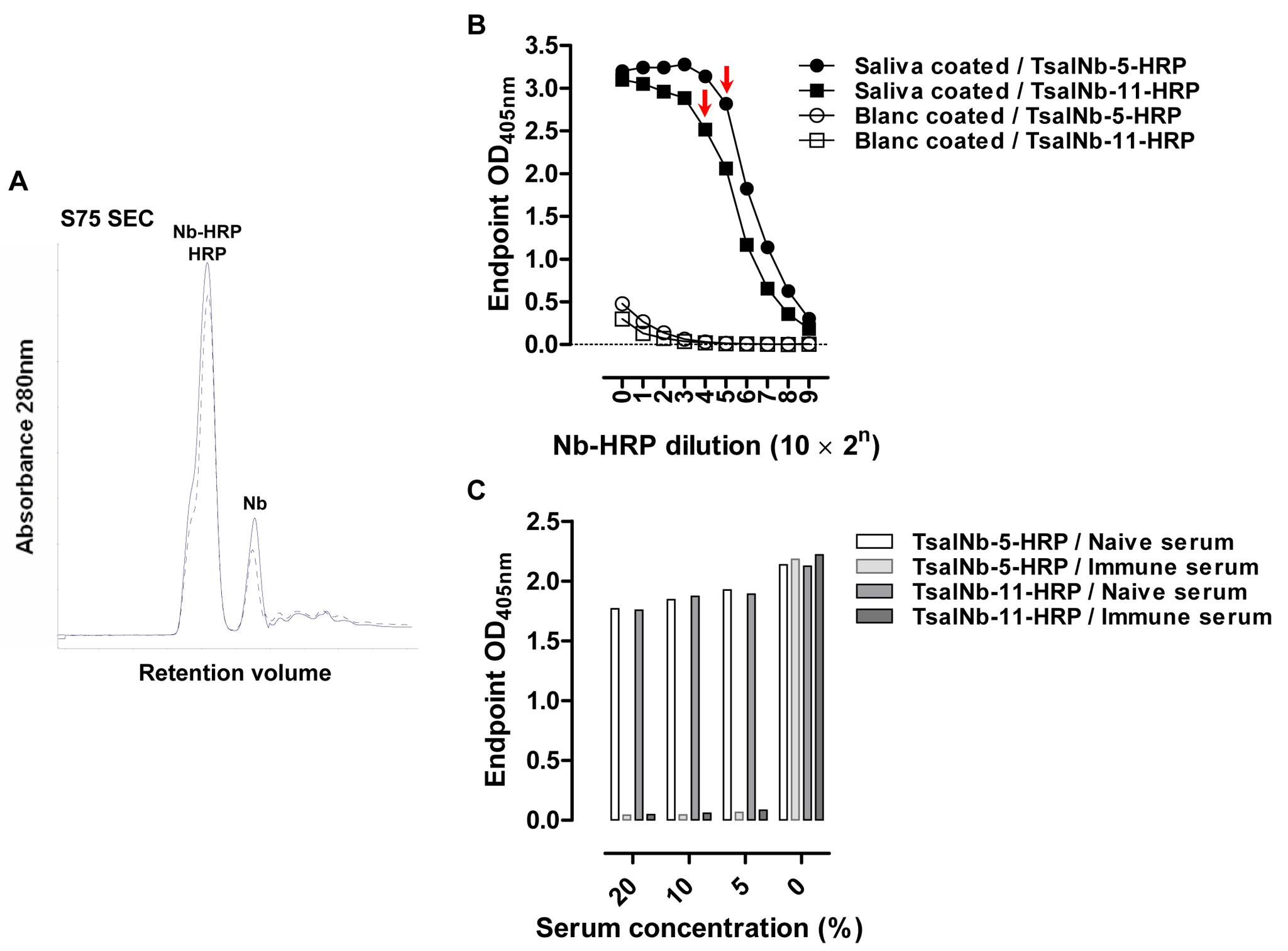

Supplement: S1 Fig — (A) Size exclusion chromatography to exclude unlabeled Nbs from the TsalNb-5 (solid line) and TsalNb-11 (dashed line) HRP-conjugation reactions. (B) Titration of TsalNb-5-HRP and TsalNb-11-HRP and binding (endpoint O.D.405nm) onto G. m. morsitans saliva coated and blanc coated wells. TsalNb-HRP working dilutions for subsequent tests were chosen at the start of the declining phase of the Nb-HRP binding curve (arrows). (C) Inhibition of TsalNb-HRP binding following incubation of the G. m. morsitans saliva coated wells with several dilutions of tsetse-exposed immune or naive rabbit serum. (TIF) [file pntd.0003456.s001.tif]

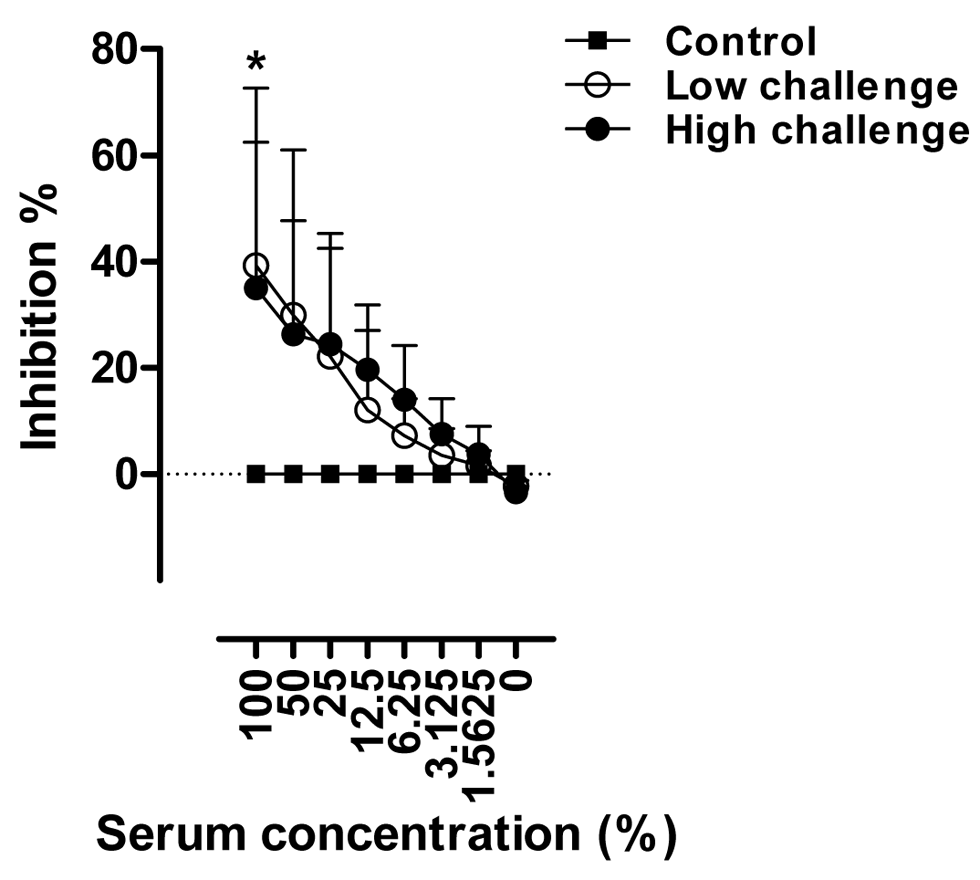

Supplement: S2 Fig — Porcine plasmas of control animals and animals exposed to a low or high tsetse challenge regimen sampled at the peak of anti-Tsal antibody responses were serially diluted and tested in the competitive ELISA. Presented data are the percentage inhibition with the means and 95% CI. Significance levels based on two-way analysis of variance are indicated in the graphs (*p<0.05). (TIF) [file pntd.0003456.s002.tif]

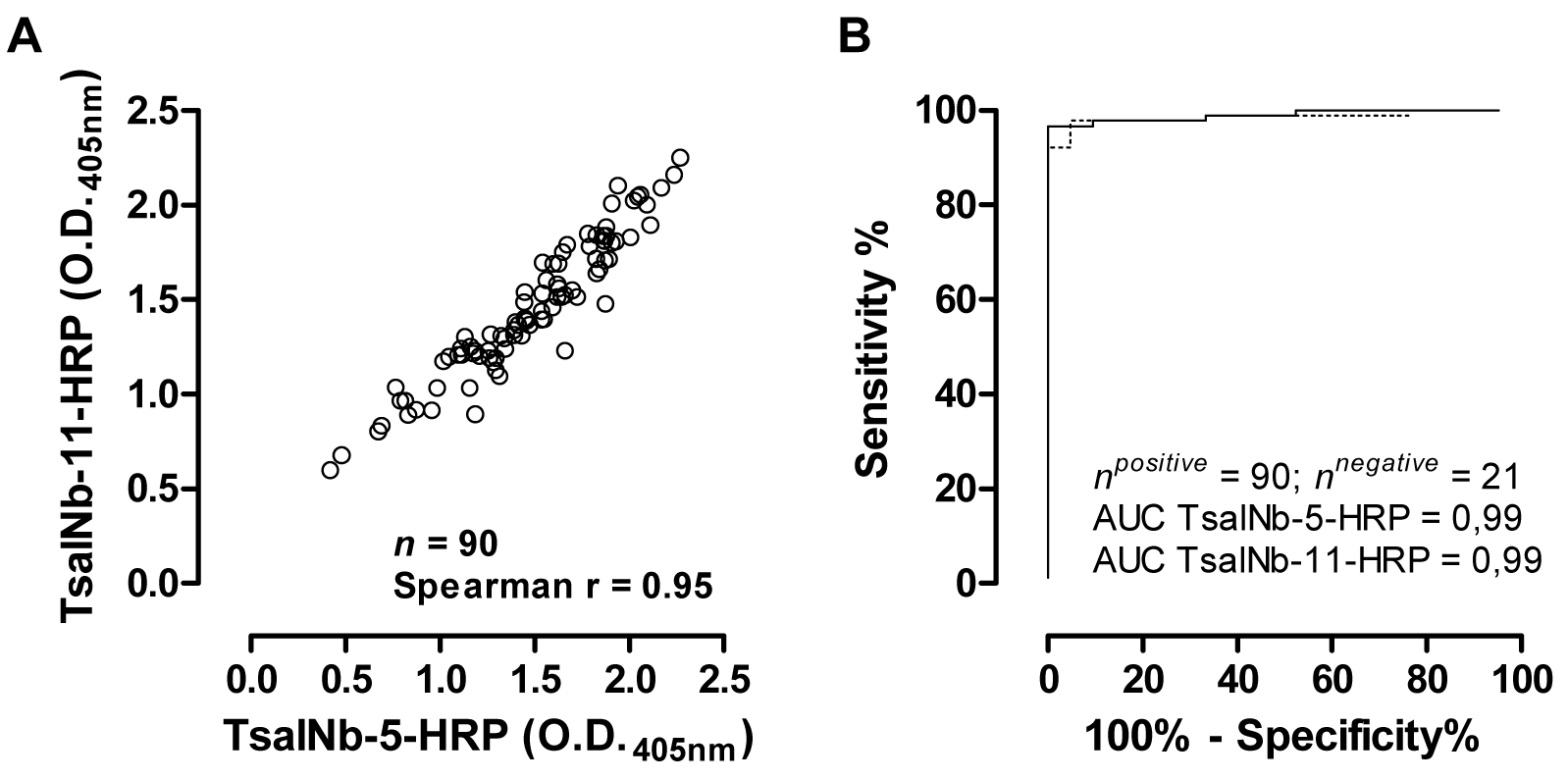

Supplement: S3 Fig — (A) Scatter plot analysis of the anti-Tsal antibody responses (O.D.405nm) detected in undiluted porcine plasma samples (n = 90) with the competitive immunoassay using TsalNb-5-HRP and TsalNb-11-HRP for detection. Overall test results with the two Nb-HRP detection moieties were compared using the non-parametric Spearman correlation test with the correlation coefficient r as output. (B) Sensitivity and specificity of the competitive assays were assessed by receiver operating characteristic (ROC) curve analysis of the O.D.405nm values of exposed (n = 90) and non-exposed mice (n = 21). The area under the ROC curves (AUC) for detection with TsalNb-5-HRP (solid line) and TsalNb-11-HRP (dotted line) are shown as a measure for the individual test performances. (TIF) [file pntd.0003456.s003.tif]
